# Reaching out and reaching up - developing a low cost drug treatment system in Cambodia

**DOI:** 10.1186/1477-7517-9-11

**Published:** 2012-03-12

**Authors:** Axel Klein, Vonthanak Saphonn, Savanna Reid

**Affiliations:** 1 Centre for Health Services Studies, University of Kent, Canterbury, UK; 2 National Institute of Public Health, Ministry of Health, Phnom Penh, Cambodia; 3 School of Community Health Sciences, University of Nevada at Las Vegas, Las Vegas, USA

## Abstract

Cambodia, confronted by the spread of drug misuse among young people, requested support from international agencies to develop a drug treatment programme in 2000. The initial plan developed by the United Nations Office on Drugs and Crime was to set up a number of conventional drug treatment centres in urban areas. During the planning phase, however, the project was redesigned as a community based outreach programme. Ten Community Counselling Teams have been formed and trained in pilot areas, and within the first year of operation 462 drug and alcohol users contacted. Comprising former drug users, family members affected by drug use and health care staff, they have drug scene credibility, local knowledge and connectivity, and a rudimentary level of medical competence. Crucially, they enjoy the support of village elders, who are involved in the planning and reporting stages. While the Community Counselling Teams with their basic training in addiction counselling are in no position as yet to either provide or refer clients to treatment, they can provide brief interventions, organise self help groups, and most importantly provide an alternative to law enforcement. By taking a development centred approach, with emphasis on community, empowerment and inclusion, it provides a constructive and inclusive alternative to medical approaches and the compulsory drug treatment centres. The paper is based on an evaluation involving interviews with a range of stakeholders and a review of project documents.

## Introduction

The prevailing policy consensus in Cambodia is that drug misuse was unknown until the mid 1990s, an unintended consequence of the country opening its borders to travellers and trade in 1993 after decades of war and isolation. Since then there has been a sharp rise in drug seizures and arrests and the availability of amphetamine type stimulants, opium, cannabis and prescription medicine [1,2].

By 1996 the country had been identified as a drug trafficking transit route [3] and by 2001 local drug consumption was being reported [4]. Patterns of use were changing, as they had done earlier in Vietnam, with a shift from opium to heroin, and the rising popularity of amphetamines [5,6]. In 1995, the government formed an inter-ministerial agency, the Secretariat of the National Authority for Combating Drugs (NACD) and solicited support from the international community. The UNODC quickly established itself as one of the key partner agencies, together with WHO, UNAIDS and bilateral agencies [7]. It solicited donor funding to support the NACD financially, assisted the government with the drafting of drug control legislation, and clean-up operations following the discovery of a clandestine laboratory.

The need for drug treatment was recognised in early 2000, and led to the formation of an illicit drug-related HIV and AIDS working group (DHAWG) by the NACD and the National Aids Authority (NAA). Without a coherent strategy, problem users and their family were relying on the services of Buddhist monasteries, with a long tradition of taking in troubled young men including drug users, and the patchy service provision by a handful of NGOs like Friends International, the Khmer HIV/AIDS Alliance (KHANA), Khmer Youth Association and Cambodia Network of Positive People. Too often the effectiveness of these service providers was hampered by a lack of critical understanding of substance use, behaviour change or harm reduction [8,9].

Many medical practitioners at the time had reservations about treating drug addicts, afraid of drug dealers bursting into the clinic to retrieve their clients. It was left to the police and army, therefore, to set up the first residential centres in 2003. In the absence of support from the Ministry of health, clients were left in the hands of untrained staff who applied a regime of physical discipline and isolation of very limited therapeutic benefit. By 2010 there were 14 of these facilities, each catering for between 60 - 100 people, who would be compelled to stay for up to six months. Nominally free of charge, in practice clients or their family were expected to contribute.

Former clients and family members were soon disappointed with compulsory drug treatment, as conditions were often brutal [10,11] and the methods ineffective. Clients exiting these centres reported a high relapse rate [12]. A number of NACD officials became therefore concerned that "drug treatment centres do not have treatment as a focus". International technical experts working in the field sought to address this gap by developing medical treatment services with support from the international community. UNODC was well placed as the lead agency, given its role as conduit of funding to the NACD and its technical expertise. A project was formulated with the objective to "increase the capacity of Cambodian healthcare professionals, both at the governmental and non-governmental level, to respond to the needs of people using illicit drugs, through development of coordinated, community-based drug abuse counselling, treatment and rehabilitation care programmes" [13]. Originally, the idea had been to set up several centres employing different treatment modalities and then identify the most effective method for Cambodia. With the arrival of a new project manager, however, the project focus shifted from medical intervention to community outreach. This sparked controversy among stakeholders, and caused the project to fail against its erstwhile objectives. Against the bleak backdrop of military run detention centres and rising levels of drug consumption, the story of project failure can only add to the sense of despondency over drug policy and drug treatment in Cambodia. The purpose of this article is to seize on some of the achievements that were recorded by the project, and to note that in Cambodia as in other parts of Southeast Asia moralistic and repressive approaches to drug control are often balanced by a strong sense of pragmatism [14-16].

Project H83 as piloted in ten rural and urban field sites is an example of such pragmatism and innovation. It is a bottom up, community driven approach, where skills are built up through a combination of training and experiental learning. It is low cost in terms of maintaining the field teams and responsive to service user need. This article is based on an evaluation of project achievements and field visits to two pilot project intervention sites, TonleBassac commune in Phnom Penh Capital and Rattanak commune, Battambang province.

## Methods

The evaluation was conducted over a three week period in September - October 2009 following the multi method evaluation model laid out in the UNODC guidance literature. ^a^It including the study of documents both at home and at the UNODC office in Phnom Pen, including the different versions of the project document, progress and monitoring reports, where available previous evaluations, self evaluations and client feedback, policy documents, documents from other projects by UNODC or other donors, and scientific literature. This was followed by collecting data from a range of primary sources, particularly interviews with key stake holders (both face to face and by telephone). In all thirty seven informants participated in the interviewing process, including: government officials (Phnom Penh municipal health department, NCHADS focal point, Planning and Training Department), experts from international agencies (WHO, UNAIDS), community leaders, leaders in the pilot communities (commune leaders and village chiefs), members of the Community Counselling Teams, families who had contacted the teams for help, and clients. Interviews took place in a variety of settings, including ministries and town halls, private houses, compounds, and public roads. Site visits were undertaken to two community teams in Tonle Bassac and Rattanak commune and to a treatment centre. The two evaluators, one Cambodian one international, regularly cross checked on interview findings, and shared information from interviews undertaken individually.

The objective of the research was to evaluate the project against the set criteria of relevance, efficiency, effectiveness, sustainability and impact. Project achievements were assessed by a mix of indicators, including quantitative information on the number of clients seen by the CCTs, the number of counsellors participating in training courses, expenditure, time frame, and the data collection from the community census. This was followed by qualitative assessments on the kind and quality of treatment, judgements on referral practices, the level of training and commitment of CCTs and community support. It soon became apparent that the project that was finally delivered had very little in common with the original design, giving rise to considerable controversy among international stakeholders. While the failure to meet original targets, and arguably donor expectations, was noted, the outcomes of what was eventually implemented are promising in their own right.

Owing to pressures of time, it was not possible to conduct more field visits or to interview more clients. It was regrettable that no visits to existing harm reduction facilities were not programmed in, hence the number of interviews with drug users was limited.

### Ethical concerns

The stringency of Cambodian legislation on drugs and drug use made it important that the personal details of informants admitting drug use were kept confidential. No names of the drug users and family members who volunteered for interview were therefore recorded. Working in Cambodia at a time when several international publications criticized the government sharply for human rights violations in their drug treatment gave additional impetus to flagging up both achievements and potential of this alternative approach.

### The Cambodian anti-HIV campaign

Efforts to combat drug misuse have to be seen in the context of Cambodia's campaign against HIV/AIDS. In the late 1990s the country registered the highest HIV prevalence rate in Asia [17]. An extensive campaign led and coordinated by government, supported by international agencies, and implemented by a committed public health sector achieved a remarkable change in sexual risk behaviour [18-20]. The methodology of using peer workers and targeting hidden populations like commercial sex workers and high risk groups such as military and police through outreach activities has provided an excellent model that can be adapted for health promotion relating to psychoactive drugs [21].

Drug injecting and the link between drug use and sexual risk behaviour create a natural overlap for HIV prevention and drug outreach activity. Drug workers can also learn from the experience of HIV workers in stigma reduction [22], as drug use, even though experimentation is reportedly widespread among young people, remains strongly stigmatised [23]. The key difference for outreach workers in the respective sectors lies in the relationship with the authorities. As drugs are illegal and associated with other forms of criminal activity, they attract police attention. Working with drug users therefore requires inter-agency arrangement to allow service providers access to clients. The intrusion of well intended legislation on HIV/AIDS prevention was reported in 2008 following the passage of the *Law on the Suppression of Human Trafficking and Sexual Exploitation*. Subsequently, brothels were closed, and sex workers taken to detention centres for 're-education'. With women being searched in the streets and any condoms found serving as grounds for arrest the campaign pushed sex workers into unsafe practices and working on the street. The campaign reversed some of the gains made in the fight against Cambodia's HIV prevalence rates, in which the policy of 100% condom use by sex workers was credited as one of the main factors [24].

There are clear parallels in Cambodian policies on drugs and prostitution with the reliance on repressive legislation to protect public morals [25], and equally, the implementation of community programmes to protect public health. The model developed under H83 was presented as meeting National Strategic Development Plan objectives of decentralization, and derived a mandate for working at community level from the National Plan on Drug Control 2005-2010. It factored in cumulative learning from health promotion programmes particularly on the transmission dynamics of AIDS to rural areas and the transfer of risk behaviour to rural areas, where populations are less well informed and undprepared [26-28].

### Evaluation findings

#### Sensitization and knowledge Gap

The project began in 2007 when the NACD coordinated representatives from local government, the ministry of health and other critical stakeholders to work out a joint plan of action. Two things were critical - the participation of commune level village heads, and the commitment of funds, channelled via the NACD and the Ministry of Health. A primary need identified at this stage was to get a sense of scale of the problem. The difficulty in getting hard data is exemplified by the discrepancy in drug prevalence figure of 6,876 reported by the NACD to the 2005 UN World Drug Report against a UNAIDS consensus figure of 40,000 and a WHO-UNAIDS supported the National Centre for HIV/AIDS, Dermatology and STI (NCHADS) size estimation of 13,000 drug users and 2500 injecting drug users [29,30].

This information requirement shifted the course of the project well beyond the treatment focused objectives listed in the project document. An Indian NGO, the TTCRF Clinical Foundation was tasked with providing training for the research team - in all some 15 Master Trainers, 24 Provincial Trainers and 240 grass root workers. This cadre of researchers, with the support of village councils, then went on to produce a baseline behavioral survey mapping drug and HIV vulnerability of 477 villages in 60 communes located in 42 districts of Cambodia. The survey reached a vulnerable population of 3,250: 1,381 drug users and 274 of their partners, 1,067 problem alcohol users and 528 of their partners. A heavy gender imbalance became quickly apparent, with males comprising 91.3% and females 8.7% of the total sample of problem users. That notwithstanding, injecting drug users (including female injectors) were identified in each survey province, giving rise to concern over service provision to very hard to reach populations who were in need. Other high risk groups reached by the survey were MSMs and entertainment workers, with many reporting concurrent drug and alcohol dependency.

The data, gathered by local enumerators and collated at provincial level, provided the basis for the next phase of integrating a bottom-up as well as a top-down approach. The survey established the reality and scale of drug use for both local and national level stakeholders. The training on rapid situation assessment and undertaking peer led interventions, providing low cost care and support along with safer practices, as well the actual experience of carrying out the survey also empowered a cadre of commune level researchers and council elders supervising the process with the confidence to move forward. In each commune teams were formed, comprising of health workers, often recruited from health centres, former drug users and the partners or family members of former or current users. Through this composition the teams combined a level of medical competence with drug scene credibility and reach.

#### Community counseling teams

Then followed the training of Community Counseling Teams (CCTs) in basic drug knowledge and undertaking interventions. Some 42 staff from the city and district referral hospitals, health centres and CCT team leaders attended MOH facilitated training on Screening, Brief Intervention, Stages of change counselling, and detoxification. They also received information on drug dependency and HIV, drug-drug interactions (between street level drugs, prescription drugs, anti-TB drugs, ART), and substitution therapy. After the primary cadre of CCTs had been trained and facilitated by the NACD, subsequent trainees were placed with existing teams. The core teams were also eligible for refresher courses and further training on case record documentation and recording of referral data.

The work of the CCT was designed as a part time 'voluntary' activity as team leaders being health practitioners with other commitments could only make limited time available. Former users and family members had fewer formal sector employment commitments- "the criteria for selection being educational equivalence with drug users in the survey communes and current unemployment, so as to foster a sense of usefulness and dignity among members of a stigmatized population [Interview with Project Coordinator]." In the process that followed, every single CCT team visited five pilot villages most affected in a commune up to 10 times a month. Each village is visited once a week always on the same day and usually at the same time as when drug users are known to congregate. Once a drug user has been contacted the team have to find a place to work that guarantees a modicum of privacy.

It could be a room client's family home, a community building, the health centre, or an open space on the edge of the village. In the initial meeting the CCT conducts an 'assessment' of drug and alcohol use, as well as a range of other conditions. This may include voluntary HIV testing, and STI and TB referral for screening services to the health sector. Following the assessment, the team then engages in what they call 'counselling', but what is basically a dialogue where the client is invited to talk about his personal life, problems and drug use, while the CCT inform him of health risks, legal consequences and alternatives. To quote a CCT Team Leader who is a member of the Health Centre linked to one of the pilot communes -"When we go down we meet the family or partner of the user and provide information and support. The aim is to change the behaviour of the user, but it is entirely voluntary, so we are different from the police." He goes on to say that this seems to be the right approach at an early stage in a client's drug use, though heavier methods may be required later on [Interview, 09/09/09 Tonle Basset].

It is essential for the CCT team to build rapport with the drug users and get comfortable with their work. They have created a space in which people can safely discuss their drug use and explore actual or potential problems. It has also introduced new perspectives on an issue dominated by crime control discourses. Most remarkable is the innocence and lack of information available to young people in rural areas [31]. The route into problematic use was simply laid out by one young informant who started using yama "because it makes me happy and 90% of young people here were using it too [Interview with former drug user (1) in Rattanak commune]."

The two functions of data collection and counseling are often merged into one. One team in Rattanak Commune reported that the initial purpose of an interview could be to collect information but then evolve into an intervention. As they started asking about drug and alcohol use word about their work spread. Subsequently families or partners would call them to come and talk to someone with drug and/or alcohol issues. The motivation of team members varies. One young woman reported that her brother was in a re-education facility and that she wished there was an alternative available locally for him and others like him. One former user said "I want to give something back. If I have a chance to tell people that drugs are bad then I will." Each CCT member is remunerated with around $500 a year to cover expenses.

The work is spearheaded by the CCTs, but it is essential to remember the communal context. In villages and communes the work is supported by chiefs and elders. One village chief explained his support for the project. "The drug users are our children, we should not send them to the police, they can become too violent. In the past the drug users used to hang out openly, now they don't do that anymore [Interview with former drug user (2) in Rattanak commune]". This confirmed claims by CCTs that drug users were less visible as a consequence of their outreach work, and perhaps even reducing their consumption.

## Discussion

When comparing project results against the project objectives as listed in the project document, the discordance between original plan and actual achievement becomes apparent.

Output 1: Hold a meeting of all relevant stakeholders and establish a Working Group to discuss the best way to develop a drug abuse treatment and rehabilitation system in Cambodia.

Output 2: Establish Drug Abuse Services that will provide both "open access" services, such as outreach and counselling, as well as "structured" treatment and rehabilitation services to people at-risk from the habit of using illicit drugs.

Output 3: Improve capacity of service providers so that they can respond to drug abuse issues through providing coordinated, community-based counselling, outreach, treatment and rehabilitation services to existing drug abusers, vulnerable groups and their families.

Output 4: Establish models of community based best practice drug abuse outreach, counseling, treatment and rehabilitation approaches and techniques for effective implementation within the socio-economic environment in Cambodia

Output 5: Increase number of people reached through outreach, counseled, treated and rehabilitated from the abuse of drugs.

Output 6: Adopt selected best practices portfolio of medical, psychiatric and social work training courses and curriculum for community-based drug abuse outreach, counselling, treatment and rehabilitation into Cambodian Drug Abuse Service Network.

The project had originally been designed to develop a drug treatment model appropriate for Cambodia, and to adopt the experience for training purposes. This has clearly not been achieved, leaving the country without a critical service at a time of rising need. The reorientation of the project towards a community based intervention, and the reformulation of the project output to "select[ing] the best practices of service and training protocols [32]", therefore constitutes a missed opportunity. Moreover, it was hoped that the treatment facility would have provided a showcase for the compulsory detention centres and brought about a change in their current practices. Whether this would have really been feasible is now a matter of speculation, all that is left to say is that the original objective of setting up a high quality treatment facility has not been realized.

The project is equally ineffective at referring clients to a treatment centre, for the simple reason that there are none to speak of. Though 'referral' hospitals figure at the top of the project flow chart as the ultimate destination for project clients, they do not as a rule, take in problem drug users. The CCTs have no formal relationship with the compulsory detention centres, which are run by different ministries and have no link with either health or local government.

While it has succeeded in reaching a number of people and has established community based activities, these constitute neither treatment nor counseling. There certainly has been a rise in 'outreach' activities, but it is not clear how these can be squared with project objectives. When measured against the project objectives and purpose, the project has been clearly unsuccessful.

Yet, the project has generated a lot of activities on the ground and scored some results that were not in the original project plan. They could be gathered under the umbrella of harm reduction and include the development of a non custodial alternative for drug users, brief interventions, outreach, the reintegration of former users, information gathering and promoting the idea of evidence based policy, and educating local elders about drug issues.

Local structures were being laid with the 10 Community Counseling Teams, 12 health centers and 9 referral hospitals (RHs) connected to 50 villages in 5 provinces. By September 2009 some 462 drug and alcohol users had been reached and 100 affected family members were followed up. Among all drug and alcohol users reached by the program at the time of evaluation in September 2009, 62 had been lost to follow up during this period, given a 13.4% of lost to follow up rate.

What is equally clear, however, is that the activities by the CCTs cannot possibly be described (as yet) as drug or alcohol treatment. They are better described as brief interventions supported by HIV testing or harm reduction. Referral to treatment cannot be expected if setting up such a facility was the original project purposed that was traded in for a low threshold outreach service. There is considerable reluctance among the families of many to refer children or partners to military run re-education centres, but even those willing are reported to be deterred by the relatively high cost. While some of the centres are officially at least, free of charge, this is not the perception on the ground.

For many Cambodian drug users, however, including those committed to compulsory drug treatment centres, the need for residential care is in any case questionable. The main drug of choice are ATS, known as Yama, a substance not associated with medically risky detoxification. The interventions by the CCT, however rudimentary by the standards of advanced psychotherapeutic practice, may still have a valuable effect in terms of behavior change. The CCTs interviewed, as well as family members claimed that their work had brought about improvements in terms of reduced drug use, and there are indications from other public health campaigns that even brief interventions can positive health outcomes [33].

The biggest improvement, however, is in the wholly unintended project achievement in terms of harm reduction. The very activity of CCTs has resulted in less police activity because at local government and at commune level policing and CCTs are coordinated. Moreover at local level police officers have eased off their drug enforcement when CCTs are active. This has reduced the numbers of arrests and criminalization of, particularly, young drug users. The deputy governor of the province of Battambang said that the CCTs were doing an excellent job because it meant that drug users were not being sent to treatment but kept in the community.

Involving former drug users in the CCTs, lends a level of credibility that other agencies working in the field are so sorely missing. It also provides ex-users with a livelihood and a purpose where their experience can be put to a positive use.

The project emphasis on data gathering via household surveys in the pilot communities has also achieved a number of valuable benefits quite outside the ambit of the original project purpose. First, it reinforced the idea of evidence based policy making while at the same time skilling up teams of researchers, data inputters, and analysts. It also created a linkage in data transmission from the commune, to local government and up to NACD headquarters.

The involvement of the community at every stage, from data gathering, to establishing the CCTs, and feeding back on progress, has also had a number of results. Community elders, like village or district leaders, were empowered to participate in an urgent and contemporary policy issue. And by getting involved in the research first, and the work of the CCTS later, they got a better understanding of an issue that is often difficult to understand by older generations.

## Conclusion

There is a confluence of national and international opinions that drug misuse and the dependency it causes in the user are a medical problem, which require a medical intervention. According to the US National Institute for Drug Abuse: *"Addiction is a chronic disease similar to other chronic diseases such as type II diabetes, cancer, and cardiovascular disease. No one chooses to be a drug addict or to develop heart disease" *[34]. Compared to the punishment focused moral condemnation of drug misusers that dominated drug control discourses for many years and is still extant in some countries, especially in East Asia, the shift to the medical model is both humane and persuasive. Only, it has to be remembered, that drug problems are not quite medical issues like diabetes, cancer or heart disease. After a generation of research we are no nearer discovering the aetiology of drug misuse, have little understanding of the disease course, can neither make prediction over outcomes nor offer definite cures. The high failure rate among addicts exiting treatment has prompted treatment specialist to re-define addiction as a *relapsing *condition. While residential treatment may effect detoxification, the challenge of staying drug free has shifted the intervention focus in many countries from treatment to social re-integration and maintenance. The need for holistic support packages and the prominence of psychotherapeutic interventions has engendered a definition of addictions as a bio-psycho-social condition.

In the context of developing countries, with often shallow histories of drug misuse, recent discussions have reframed drug misuse issues as development impediment [36]. Adding a new dimension to the medical, public health and crime control considerations, has created both tensions and opportunities. Health care models incline towards high end service provision like residential treatment, medically assisted detoxification, and opiate substitution, with patient well being and clinical excellence as the guiding principles. By re-situating drug control within a development context, new principles such as empowerment, inclusiveness, and grass roots development move into the foreground. The experience of the UNODC CMBH83 project in Cambodia is a valuable experience for other developing countries. These can be captured under the following headings

1. Reach and consistency - working at commune level means that the intervention can be easily rolled out across the country and that interventions are carried within each community by workers with local knowledge to the most vulnerable risk group. As the staff are local the activities are not one off campaigns but make for a regular relationship with positive knock on effects beyond the original intervention

2. Cost effectiveness and sustainability - at $1,500 per CCT the project can be maintained beyond the life of the original project and over successive budget cycles; it provides a local level delivery that is cheaper than both health centre or hospital care and is much cheaper than criminal justice interventions. The start-up costs were much higher because of the training, but particularly the administrative and management costs. Where international agencies are involved large amounts are eaten up by salary, overheads and travel. But once running, projects can be kept running on a shoestring.

3. Holistic interventions - the complexity of substance misuse, with its various medical, social and economic components, plays into the hands of a local CCT with mix of skills, personal commitment and community backing. Clients enjoy the benefits of counselling as well as social reintegration, as the teams are embedded in the community and supported by village elders.

Valuable as the experience of the project has been in laying the foundation for a comprehensive early intervention system there are a number of issues that will have to be addressed in coming years. At the point of evaluation the work conducted by CCTs was not drug treatment but at best a brief intervention. While the level of training of team members was adequate for dealing with young amphetamine users it is important that skills are upgraded and a system of professional development be put in place.

Five years after the UNODC programme began there still is no drug treatment centre to speak of in Cambodia - there are several re-education centres, and places where drug addicts are sent to reform but nowhere fittingly described as treatment. The NACD with help from the international community should continue in its endeavours to set up a network of low cost treatment facilities. In recent years a number of NGOs have begun providing treatment, but only one, MithSamlanh, does so on a voluntary, no charge basis.

Devising an evidence based approach with village level researchers and provincial level data collation ensured that stakeholders a different levels had an insight into and then took ownership of first the problem and second the intervention. Yet the data itself des not seem to be used for policy making but is passed from agency to agency in a four-tier reporting system from commune to district and to province and then on to NACD, MOH and the project office. This waste of energy will breed resentment if not rectified.

It is too early to say how successful the project will be in stemming the spread of illicit drugs across Cambodia. Yet, the approach of providing a web of teams with a mixed role of rudimentary treatment, prevention and reintegration of former users, is innovative, ambitious and promising. There are key benefits, such as including many stakeholders and empowering local communities, and most critically avoiding the unintended consequences of criminal justice interventions.

Community activities need to be part of a broader spectrum of policy, and depend on ongoing investment in staff and infrastructure by central government agencies. Skill levels have to be upgraded and referral centres are yet to be created for the scheme to achieve full potential. Already, however, the project with its village level data gathering, outreach and community backing provides a model many other developing countries may wish to look into.

## Endnotes

^a ^UNODC, 2008. *Evaluation Handbook: A practical Guide for use by UNODC Staff to plan, manage and follow up an evaluation*. Vienna: UNODC, Independent Evaluation Unit.

## Competing interests

The authors declare that they have no competing interests.

## Authors' contributions

The research for this article was carried out by AK and SV; the article was written by AK 75%, by SV 20% and by SR 5%. All authors read and approved the final manuscript.
